# A critical evaluation of the algorithm behind the Relative Citation Ratio (RCR)

**DOI:** 10.1371/journal.pbio.2002536

**Published:** 2017-10-02

**Authors:** A. Cecile J. W. Janssens, Michael Goodman, Kimberly R. Powell, Marta Gwinn

**Affiliations:** 1 Department of Epidemiology, Rollins School of Public Health, Emory University, Atlanta, Georgia, United States of America; 2 Woodruff Health Sciences Center Library, Emory University, Atlanta, Georgia, United States of America; Walter and Eliza Hall Institute of Medical Research, Australia

The influence of scientific publications is increasingly assessed using quantitative approaches, but most available metrics have limitations that hamper their utility [1]. Hutchins and colleagues recently proposed the Relative Citation Ratio (RCR) [2], which compares the citation rate of an article against the citation rate that is expected for its field. The metric is an attractive and intuitive solution to indicate whether an article is cited more or less frequently than its “peer” publications. As a ratio of rates, RCR is an article-level metric that is field and time independent and strongly correlated with peer review evaluations [2]; the metric was central in the proposed (and withdrawn) grant management policy of the National Institutes of Health (NIH) [3] even though the RCR has been criticized for lacking a theoretical model, having insufficient transparency, and having poor correlation with other peer evaluations [4,5]. We analyzed the algorithm behind the RCR and report several concerns about the calculation of the metric that may limit its utility.

## The numerator

The numerator is the article citation rate (ACR), which is calculated as the number of times the paper was cited divided by the number of years since publication. This ACR has 2 undesirable properties: it is expected to decrease for older publications and may be too unstable for recent publications.

Research articles generally lose their relevance when science progresses, and after a while, they may no longer be cited [6]. Because the number of years since publication continues to increase, the ACR will inevitably decline, and so will the RCR. For older articles, RCR reflects their “average” influence, not the influence they may have had in their heyday. The authors reported that decreases in RCR were unlikely, but this issue was investigated over a 2-year period only. Such short-term devaluations are unlikely, but the decrease in RCR may be substantial over longer periods of time, especially for studies of rapidly evolving technologies and drug therapies. This decrease may have adverse consequences when RCR is used to evaluate the portfolios of established and midcareer researchers for whom the assessment of their current influence may be devaluated by the decreased RCRs of their older work.

For recent articles, ACR may be too dependent on external factors. For example, ACR may be inflated when articles are published ahead of print, such as through advanced online publication and preprint archives. Preprint availability increases the (cumulative) number of citations when the year of print differs from the year of online publication [7]. Similarly, articles published at the beginning of the year may have a higher ACR than those published at the end. The authors do acknowledge that RCR should be interpreted with caution within 2 to 3 years after publication or when there are fewer than 5 citations (p. 17, [2]), but these thresholds may not be high enough. Dividing the number of citations by 1 instead of 2 years doubles the RCR, and a 1-year difference in publication date still increases RCR by 20% after 5 years. Five citations will create a small co-citation network of only about 150 articles that may not be an adequate and reliable representation of the field.

Not calculating the RCR for recent papers and those with few citations seems a simple solution, but it may have major implications for the calculation of the average RCR of researchers’ portfolios. When articles without RCR are not considered, the average RCRs are overestimated. We observed that NIH’s online tool *iCite* (https://icite.od.nih.gov/) does calculate the RCR for papers published as recently as 2015 and for articles with fewer than 5 citations and includes those articles in the calculation of the average RCR. The handling of these recent and lowly cited articles in portfolio RCR calculations warrants further research to ensure fair comparisons of researcher performance.

## The denominator

The denominator is the expected citation rate, which is the normalized field citation rate (FCR). FCR is in turn defined as the average of the journal citation rates for articles that were published in the same field. We have concerns about the validity of the field definition, the calculation of the FCR, and the FCR normalization procedure.

**Fig 1:**
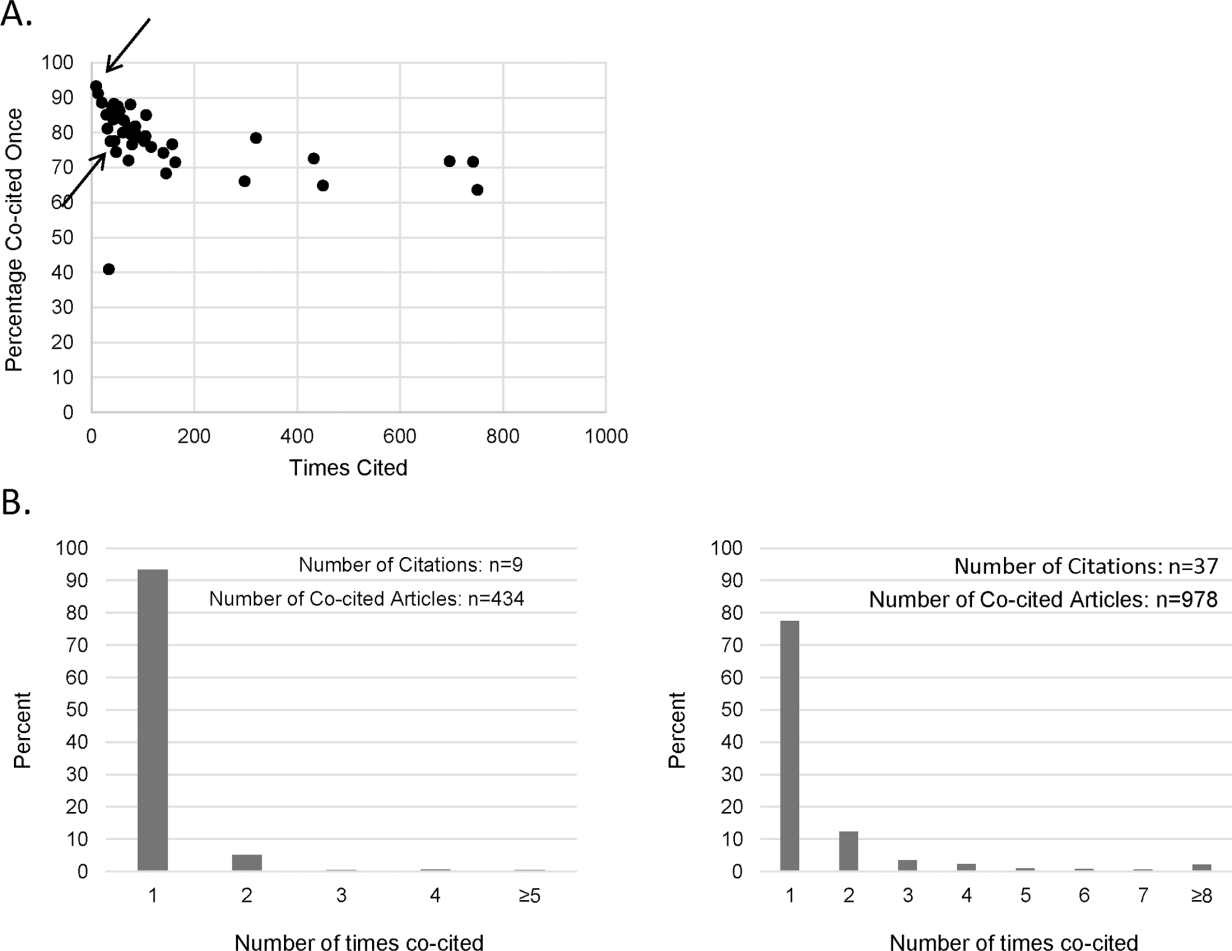
Percentage of articles that are co-cited only once. Data were obtained from our review of 42 meta-analyses [9]. Co-citation analyses were conducted for the highest-cited article (max 1,000 citations) included in each meta-analysis. (A) Percentage of articles in the co-citation network that were co-cited only once with the highest-cited article. (B) Distribution of co-citation frequencies for 2 articles (indicated by arrows in Fig 1A). The number of co-cited articles indicates the size of the co-citation network. Data are provided in S1 Data.

First, the field of an article is defined as its co-citation network, which includes all articles that have been cited together with the article [8]. This flexible definition is preferred because it is expected to better represent interdisciplinary research. The problem with this approach is that it includes many articles that have little to do with the topic or the scientific field of the publication in question. Our research showed that frequently co-cited articles were indeed on the same topic as the research article, but most “miscellaneous” co-citations were not [9]. We observed that on average 80% of articles were co-cited only once, irrespective of how frequently the article was cited (Fig 1A). A more valid field definition would consider only frequently co-cited articles or at least only those co-cited more than once, but this would lead to a pronounced reduction in the number of co-cited articles (Fig 1B) and generate unstable RCR values even for articles with a reasonable number of citations.

Second, FCR is the average citation rate of the journals in which the co-cited articles were published, but this journal citation rate (JCR) is not defined in the article of Hutchins et al. We read that “almost all JCRs are quite stable over time (S1 Fig; S1 Table)” (page 5, [2]) and found that both the figure and table only present journal impact factors (JIFs), which the authors acknowledge in both titles (“JIF stability over time…”). The authors also write that they used the “2-year synchronous JCR,” for which they cite 2 articles about the JIF [10,11]. The first citation, Rousseau and Leydesdorff [10], presents a formula for the “n-year synchronous journal impact factor” and the authors write that this formula “when n = 2…obtains the classical Garfield (1972) journal impact factor.” The classical Garfield paper was the second article about the JIF cited by Hutchins et al. [11]. The denominator of RCR thus essentially is a normalized average JIF. Interestingly, Rousseau and Leydesdorff also give a formula for the “n-year diachronous journal impact factor,” which takes all articles published in the same year and follows over time how many citations they accrue. This diachronous impact factor is a JCR and equivalent to the average ACR of all articles published in the journal in a specific year.

A 2-year JCR, whether synchronous or diachronous, assesses the citation rate for articles in their first 2 years after publication. Using the JIF in the denominator thus assumes that the expected citation rate is constant over time and remains at the same level as in the first 2 years. This may be a reasonable expectation for the calculation of the RCR for recent publications, but not for older articles [6]. Using unrealistically high expected citation rates will underestimate their RCR, which again will negatively influence the average RCR of established and midcareer researchers.

Third, the RCR is normalized against a collection of 311,497 publications from NIH projects to obtain a benchmark against which articles can be compared. Through normalization, the average RCR is set at 1 to facilitate interpretation of the measure as it applies to an individual paper. The problem with normalization is that it may mask problems with the definition and calculation of the denominator. Normalization could be justified to adjust the RCR values if their actual (not normalized) mean is not exactly 1. A regression model that accomplishes such minor adjustment will have an intercept that is close to 0 and a slope close to 1. The coefficients of the regression models used to correct the mean RCR had intercepts ranging from 0.12 to 1.95 and slopes from 0.50 to 0.78, depending on the year of publication of the co-cited article (Supplementary Table S2 [2]). This demonstrates that a substantial adjustment was needed to get the mean RCR to 1, which raises questions about whether the average JCR is a suitable proxy for the FCR. Furthermore, the model regresses FCRs to “the mean” and increases the variation in RCR: higher RCR values become even higher, and lower RCR values become lower. The need for a massive adjustment and the possible unwanted effects on RCR values need to be understood before it can be concluded that normalization is justified.

## Concluding remarks

An article-level influence metric needs to be valid across its entire range of values and to be meaningful for its major proposed uses, such as assessing the productivity and influence of individual researchers and programs. The RCR may not meet those criteria. The inaccurate definition of the field, the use of a 2-year JCR as the basis of the expected citation rate, and the substantial adjustments entailed by the normalization process all raise doubts about the validity of the metric. In addition, the reduced influence of older articles and the inclusion of recent and lowly cited articles raise questions about the validity of the average RCR for researcher portfolios. The RCR could be an attractive and intuitive metric, but the reported concerns warrant further research for reliable and valid fixes before the metric can be implemented in grant management policies.

## Supporting information

S1 Data Data that were used to make Fig 1. (XLSX)

## Abbreviations

ACR: article citation rate
FCR: field citation rate
JCR: journal citation rate
JIF: journal impact factor
NIH: National Institutes of Health
RCR: Relative Citation Ratio
